# Full Neutralization of *Centruroides sculpturatus* Scorpion Venom by Combining Two Human Antibody Fragments

**DOI:** 10.3390/toxins13100708

**Published:** 2021-10-06

**Authors:** Lidia Riaño-Umbarila, José Alberto Romero-Moreno, Luis M. Ledezma-Candanoza, Timoteo Olamendi-Portugal, Lourival D. Possani, Baltazar Becerril

**Affiliations:** 1 CONACYT, Instituto de Biotecnología, Universidad Nacional Autónoma de México, Apartado Postal 510-3, Cuernavaca 62250, Morelos, Mexico; lidia.riano@ibt.unam.mx; 2 Instituto de Biotecnología, Universidad Nacional Autónoma de México, Apartado Postal 510-3, Cuernavaca 62250, Morelos, Mexico; jose.romero@ibt.unam.mx (J.A.R.-M.); lmlecan832@gmail.com (L.M.L.-C.); timoteo.olamendi@ibt.unam.mx (T.O.-P.); lourival.possani@ibt.unam.mx (L.D.P.); 3 Instituto de Biotecnología, Departamento de Medicina Molecular y Bioprocesos, Universidad Nacional Autónoma de México, Apartado Postal 510-3, Cuernavaca 62250, Morelos, Mexico

**Keywords:** *Centruroides sculpturatus*, human scFv, venom neutralization

## Abstract

A fundamental issue of the characterization of single-chain variable fragments (scFvs), capable of neutralizing scorpion toxins, is their cross-neutralizing ability. This aspect is very important in Mexico because all scorpions dangerous to humans belong to the *Centruroides* genus, where toxin sequences show high identity. Among toxin-neutralizing antibodies that were generated in a previous study, scFv 10FG2 showed a broad cross-reactivity against several *Centruroides* toxins, while the one of scFv LR is more limited. Both neutralizing scFvs recognize independent epitopes of the toxins. In the present work, the neutralization capacity of these two scFvs against two medically important toxins of the venom of *Centruroides sculpturatus* Ewing was evaluated. The results showed that these toxins are recognized by both scFvs with affinities between 1.8 × 10^−9^ and 6.1 × 10^−11^ M. For this reason, their ability to neutralize the venom was evaluated in mice, where scFv 10FG2 showed a better protective capacity. A combination of both scFvs at a molar ratio of 1:5:5 (toxins: scFv 10FG2: scFv LR) neutralized the venom without the appearance of any signs of intoxication. These results indicate a complementary activity of these two scFvs during venom neutralization.

## 1. Introduction

*Centruroides sculpturatus* Ewing scorpion (*C. sculpturatus*) is one of the toxic species of North America that is distributed in the United States (Arizona, California (southeastern border), Nevada (southern border), New Mexico (western border), and Utah) and along the border with the Mexican state of Sonora [1]. In the United States, it is considered responsible for the majority of envenoming cases [2], with an incidence of approximately 9000 cases per year [3]. Regarding toxicity, it is the least toxic of the species evaluated so far, with an LD_50_ of 22.7 µg/20 g of mouse [4]. A recent characterization of this venom showed the presence of two main toxic components named CsEM1a and CsEd with abundances of 8% and 1.6%, respectively [5]. Like other toxins from Mexican scorpions, these two also modify the activity of mammalian sodium channels [6,7,8]. Although there is an antivenom of equine origin [9], the alternative of producing an antivenom based on antibody fragments of human origin is novel, with the advantage of eliminating arduous collections and sacrifice of thousands of scorpions as well as the use of horses.

Currently, we have two neutralizing antibody fragments derived from the parental scFvs 3F and C1, isolated by phage display procedures from a non-immune human library and using Cn2 toxin from the venom of the scorpion *Centruroides noxius* [10]. They were obtained by means of several cycles of directed evolution to increase their affinity toward Cn2 toxin as well as their cross-neutralization against different toxins from Mexican scorpion venoms, such as Css2 (from *C. suffusus*), Cll1 and Cll2 (from *C. limpidus*), and Ct1a (from *C. tecomanus*) [11]. In this way, scFv LR was generated, which is capable of neutralizing Cn2 and Css2 toxins [12] as well as the corresponding whole venoms. Similarly, scFv 10FG2 was generated, which neutralizes Cn2, Css2, Cll1, Cll2, Ct1a, CeII9 toxins (from *C. elegans*), as well as the venoms of *C. noxius*, *C. suffusus*, *C. infamatus*, *C. hirsutipalpus,* and *C. spp nov*. from Cumpas Sonora, Mexico [11]. This broad cross-neutralization of these toxins by scFv 10FG2 is explained by their high sequence identity, conservation of disulfide bridge pattern, and 3D structures [13,14,15]. On the other hand, these scFvs have been widely characterized, and we know that they are monomeric proteins with T_m_s (thermal transition midpoint temperature) close to 60 °C [16]. They also show a rapid distribution in the body, which is a great advantage in cases of acute envenoming, like scorpion sting.

Taking into account all these issues, it is necessary to continue evaluating the neutralization capacity of these scFvs against other scorpion venoms of medical importance due to their potential use as part of a last-generation antivenom against various species of Mexican and North American scorpions. In this work, the neutralization capacity of the combination of scFvs LR and 10FG2 against whole venom was determined as well as their molecular interactions by Surface Plasmon Resonance (SPR) and Molecular Dynamics (MD) with the main toxic components of *C. sculpturatus* venom.

## 2. Results

**Figure 1:**
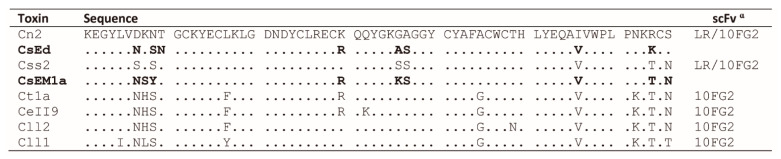
Alignment of the sequences of the toxins neutralized by scFvs LR and 10FG2 and the main toxins of *C. sculpturatus* venom (CsEd and CsEM1a in bold). Cn2, *C. noxius* toxin 2; Css2 and Css4, *C. suffusus* toxins 2 and 4; CsEd and CsEM1a, *C. sculpturatus* toxins; Ct1a, *C. tecomanus* toxin 1; CeII9, *C. elegans* toxin 2; Cll1 and Cll2, *C. limpidus* toxins 1 and 2. ^ɑ^, neutralizing scFv [11,12]. Dots indicate that these residues are conserved with respect to Cn2 toxin.

Due to the broad neutralizing capacity of scFv 10FG2 and the good affinity of scFv LR for some toxins, it was decided to evaluate their neutralizing capacity of other scorpion venoms of the *Centruroides* genus. Initially, the sequences of the main toxic components of *C. sculpturatus* venom were aligned with other toxins that are neutralized by these scFvs (Figure 1).

**Figure 2:**
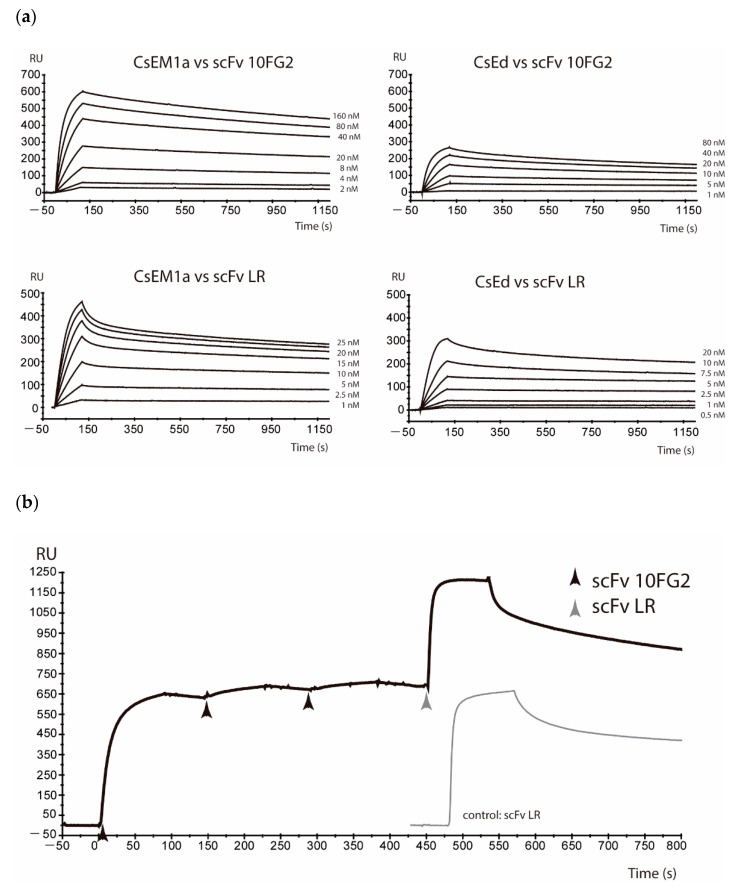
Molecular interactions in real time determined by SPR. (**a**) Sensorgrams of the interactions of CsEM1a and CsEd toxins with scFvs LR and 10FG2 at the indicated concentrations at 25 °C and with a continuous flow of 50 µL min^−1^. (**b**) Competition analyzes of both scFvs interacting with CsEM1a toxin with a flow rate of 20 µL min^−1^ and a concentration of 500 nM of each scFv. RU, resonance units.

A high sequence identity of CsEd and CsEM1a toxins (86–92%) was observed as compared with Css2 and Cn2 toxins, which are neutralized by scFvs LR and 10FG2 (Figure 1). Based on these results, the interactions of CsEd and CsEM1a toxins with scFvs LR and 10FG2 were evaluated by means of SPR in the BiacoreX equipment (Figure 2a). Purified toxins were immobilized on CM5 chips and the interactions evaluated as described in Materials and Methods. The curves of the sensorgrams allowed to confirm that indeed CsEd and CsEM1a toxins are well recognized by both scFvs. Additionally, competition assays were performed to confirm that both scFvs recognized different epitopes in these toxins. The sensorgram in Figure 2b shows that after saturating 10FG2 binding site on CsEM1a toxin, the one of LR remains available as compared to the sensorgram of control without competition. These results demonstrate that scFvs 10FG2 and LR recognize different epitopes in the toxins, as previously reported for other toxins [17,18]. Similar results were obtained with CsEd toxin (Figure S1).

The kinetic constants of the molecular interactions obtained from the sensorgrams generated at different concentrations (Figure 2a) were used to calculate the corresponding affinities. scFv 10FG2 showed similar affinities for both toxins with KDs of 1.1 nM and 1.8 nM for CsEM1a and CsEd, respectively. In the case of LR, greater differences were observed with KDs of 1.29 nM and 0.61 nM for CsEM1a and CsEd, respectively (Table 1).

### 2.1. Neutralization Assays of C. sculpturatus Venom

After the evaluation of recognition of the toxins by the scFvs, preliminary neutralization tests of *C. sculpturatus* venom were performed using the scFvs either individually or mixed (Table 2). A clear delay in the signs of intoxication was observed with scFv LR, resulting in protections of 90% and 50% of the mice envenomed with 1 LD_50_ and 2 LD_50_ of venom, respectively. These results contrast with the protection conferred by scFv 10FG2, which allowed the survival of the mice with minimal signs of intoxication when using 2 LD_50_ of venom. The neutralization assessment with 2 LD_50_ of venom showed that individually both scFvs are capable of delaying the appearance of signs of intoxication and the time of death of the animals, with a higher number of survivors as compared to the control. It was evident that scFv 10FG2 provides the best protection. A relevant observation from these results is that a mix of both scFvs at a molar ratio of 1:5 (toxin:scFv) of each one of them was capable of neutralizing 2 LD_50_ of venom without any signs of envenoming (Table 2).

As a criterion of comparison for the level of neutralization of *C. sculpturatus* venom, a series of tests were implemented increasing the number of lethal doses of venom to be neutralized by scFv 10FG2, where the amount of scFv that neutralizes 1 LD_50_ of venom (87 µg of scFv per mouse) was kept fixed for all LDs tested. The results showed that this amount of 10FG2 protects up to 5 LD_50_ (Table 3); however, some signs of envenoming were evident starting from 4 LD_50_, so it was decided to evaluate the combination of scFvs 10FG2 and LR. The results showed that this combination completely protected the mice from envenoming, which prompted us to set up a rescue test (Table 4). Here, mice were envenomed with 3 LD_50_ during 5 to 10 min before administering scFvs LR and 10FG2 in a 1:5 molar ratio (scFv:toxin) of each one. The results confirmed the complementary effect of the combination of scFvs because after 30 min from the injection of the scFvs mix, mice ate and slept in a similar way as the untreated ones.

### 2.2. Structural Analyses of scFvs 10FG2 and LR in Complex with CsEM1a and CsEd Toxins

**Figure 3:**
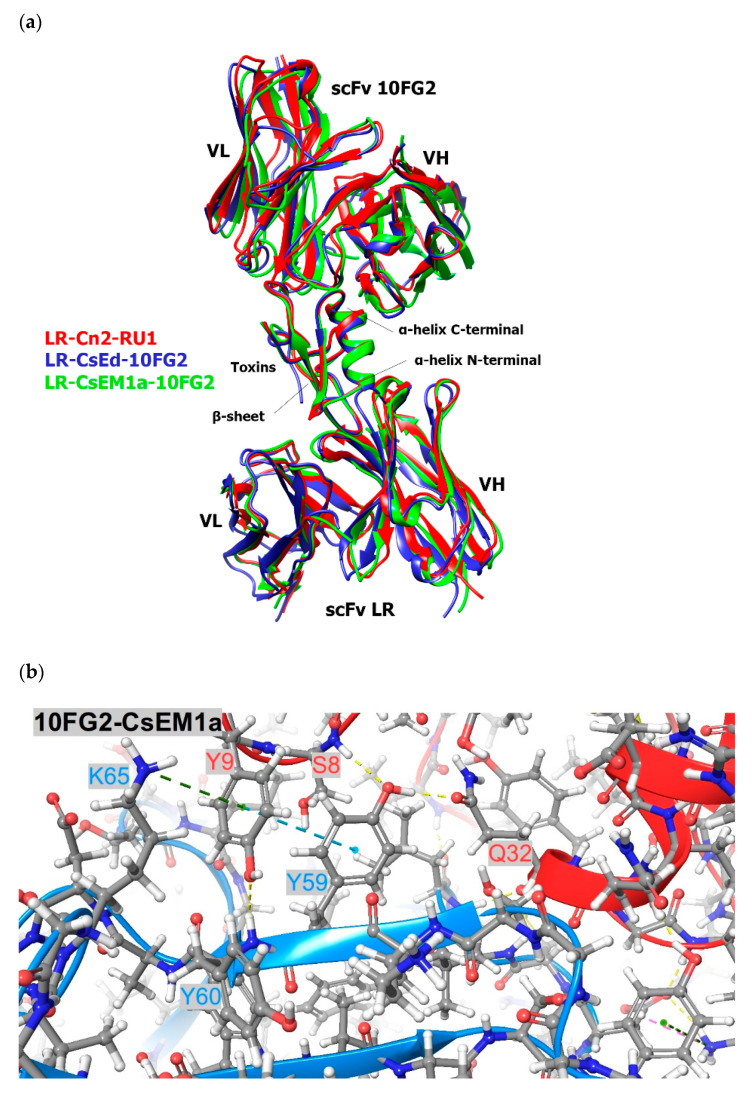

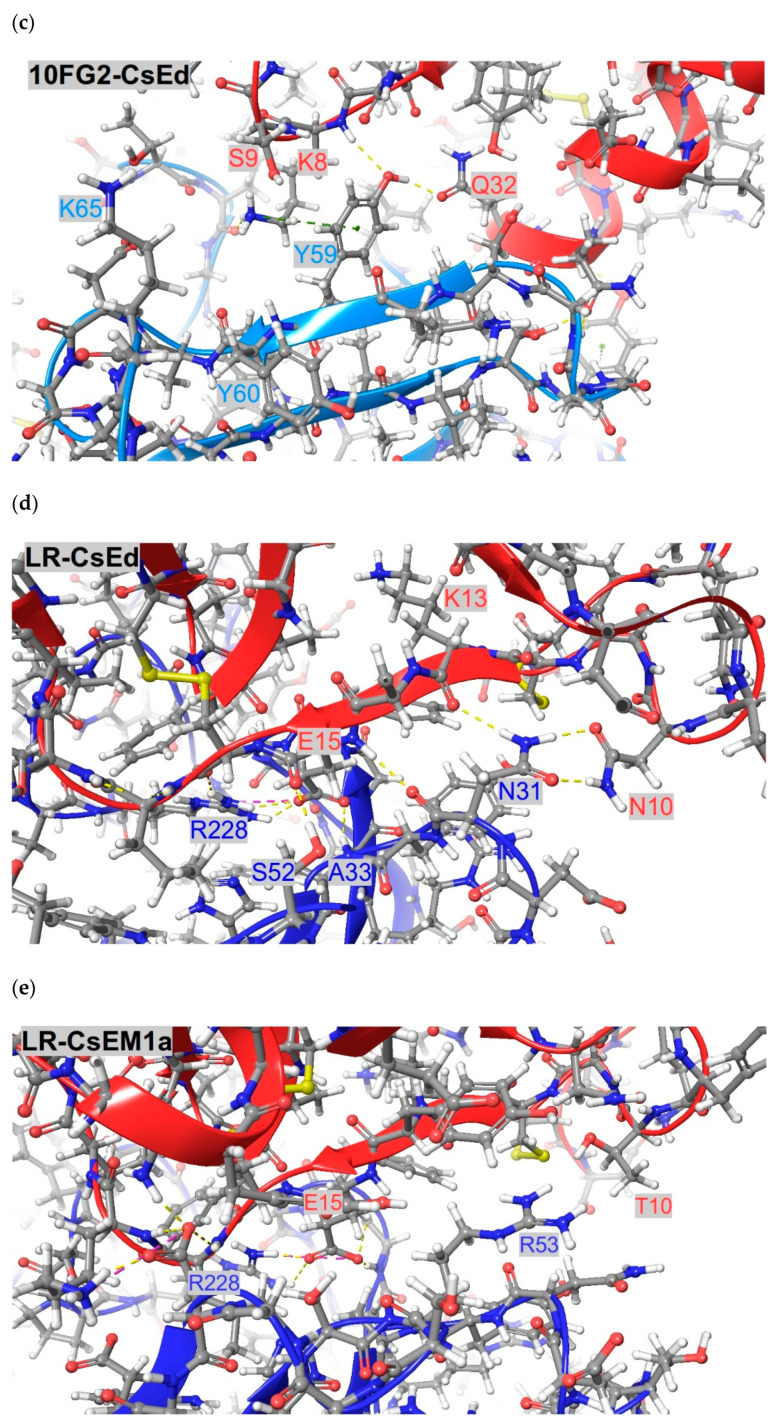
Structural analysis. (**a**) Overlaying of the structural complexes of LR-Cn2-RU1 (red), LR-CsEd-10FG2 (blue), and LR-CsEM1a-10FG2 (green). The toxins are identified by being the central structure constituted by an α-helix and three β strands (β-sheet). (**b**) Details of the interface between scFv 10FG2 and CsEM1a showing some of the residues involved in the molecular interaction. The toxin is colored in red; the scFvs are shown in blue color. (**c**) Similar details for the interactions between scFv 10FG2 and CsEd. (**d**) scFv LR-CsEd complex details in which interactions of N10 and E15 residues from CsEd toxin are indicated. (**e**) scFv LR-CsEM1a complex details at the same region of the interface shown in **d**; interactions of E15 residue are indicated.

The different types of interactions that occur at the interface of these complexes scFv-toxin were analyzed by MD (see Section 5.6). The results of these analyses are shown in Tables S1 and S2. Figure 3a shows minimal differences in the superposition of the structural models of the toxins with the two scFvs, where some of the most important contacts at the interface scFv-toxin are highlighted (Figure 3b–e). The interactions at the interface of toxin Cn2 with both scFvs were used as a control (Tables S1 and S2) since this toxin is recognized with greater affinity by both scFvs [11].

During the MD of the different complexes, the structural similarities shared between CsEM1a and CsEd toxins with Cn2 toxin were reflected in the results, as they showed that the main contacts are kept (Tables S1 and S2). These observations explain the ability of scFvs LR and 10FG2 of recognizing this group of toxins. However, there are some differences in the way that toxins interact with these scFvs at the CDRs level, which could explain the differences observed in the neutralization assays. The details of these differences can be seen in Figure 3b,c for scFv 10FG2 and in Figure 3d,e for scFv LR.

## 3. Discussion

Due to the abundance of scorpion species toxic to humans in Mexico, the optimal neutralization of venoms turns out to be a major challenge, especially if we take into account a recent estimate that proposes the existence of at least 21 toxic species in the country [1], although it cannot be ruled out that some others may eventually be identified. That is why new strategies to obtain a broad-spectrum antivenom against Mexican scorpions can take advantage of techniques such as directed evolution and phage display as well as the cross-reactivity of antibodies in order to attain the neutralization of toxins that share a high degree of sequence identity [19].

In this work, we found that the main toxins from *C. sculpturatus* venom CsEM1a and CsEd conserve epitopes similar to those of Cn2 and Css2 toxins, a reason why they are also recognized by both scFvs (LR and 10FG2). The determination of the interaction kinetic constants has made it possible to observe that all the association constants (*k*on) are of the order of 10^5^ M^−1^s^−1^, which indicates that there is a rapid binding between the scFvs and the toxins. In the case of dissociation, from the *k*off values, we were able to determine that the retention times (T_R_) show greater differences in the average binding time of the toxin-antibody complex. For example, scFv LR interaction with CsEd toxin remained for almost 71 min (Table 1), while for CsEM1a toxin, it was only 21 min. We have reported that, for a good neutralization of this epitope present in Cn2 and Css2 toxins, retention times must be longer than 250 min [12]. As CsEM1a is the most abundant toxin within the venom, and with a shorter retention time (21 min), it can be understood why scFv LR in the preliminary neutralization assays was not as efficient as scFv 10FG2. The latter recognizes a different epitope, which requires shorter retention times to be neutralized [11]. These results explain why retention times of 49 and 63 min are sufficient to neutralize the toxic effect of the two main toxins of *C. sculpturatus,* as indicated by the survival of 100% of the mice injected with the whole venom (Table 2).

On the other hand, in the neutralization tests of several LD_50_, scFv 10FG2 was capable of neutralizing up to 5 LD_50_ of venom despite using molar ratios as low as 1:2 (toxin:scFv) (Table 3), although with slight signs of intoxication. These signs were totally eliminated when using a mixture of scFvs 10FG2 and LR. It is important to note that when both scFvs interact simultaneously with a toxin, they are capable of covering around 75% of its surface [17]. This effect of covering the surface of the toxins is what would be happening with the polyclonal antivenoms produced in horses. Based on these promising results, we decided to make a more demanding evaluation of the neutralizing capacity (rescue test) [20,21] of the combination of scFvs 10FG2 and LR (Table 4). After causing a strong intoxication during 10 min with 3 LD_50_ of venom, the mixture of scFvs was administered in a 1: 5 molar ratio (toxin:scFv) of each of them. While in the control group, the death of the mice occurred between 30 min to 1 h after the injection; in the group of rescued mice, the signs of intoxication progressively diminished until mice showed normal conditions in a span of 30 min. This is the first time that we have reported a rescue using this molar ratio. These results are relevant because these assays represent a very demanding evaluation since the mixture of scFvs was not administered intravenously, where they would have been more rapidly distributed in the body of mice. However, the intraperitoneal administration used in the experiment was effective. These formats may be promising, although there are no reports on the use of scFv for disease treatment, but considering the size of the toxins (7.5 KDa), the scFv format is important due to its rapid distribution. Nevertheless, it will be the clinical trials and bio-distribution assays that will be able to confirm the advantages of this format for scorpion sting envenoming with respect to other antibody formats [22].

The results of the MD show that the wide cross-reactivity of scFvs 10FG2 and LR with the different toxins studied in this work is explained mainly through the conservation of interactions with the three toxins (Cn2, CsEM1a, and CsEd). The similarity of the sequences in the epitopes of the toxins favors the level of recognition shown by the scFvs 10FG2 and LR (Figure 1 and Figure 3a and Supplementary Materials Tables S1 and S2). However, the differences in the toxin sequences influence the affinity levels of the scFvs (Table 1). The affinity of the scFv LR for the CsEd toxin is greater than that of the CsEM1a toxin, while in the case of the scFv 10FG2, the affinity is greater for CsEM1a toxin.

The MD of scFv 10FG2 with toxins explain why the interaction with CsEM1a toxin is greater than with CsEd. Although the average number of hydrogen bridge contacts are similar (9.1 and 10.2 for CsEM1a and CsEd, respectively), there are relevant aspects to consider. Between these two toxins, there are only six differences in their amino acid sequences (see Figure 1). Of these differences, only three are at the binding interface that corresponds to the amino acids at positions 8, 9, and 10. In Figure 3b,c it can be seen that Y9 of CsEM1a establishes a variety of interactions, such as hydrogen bonds with Y60 backbone of scFv 10FG2, hydrophobic contacts with Y59 and Y60, an aromatic-aromatic interactions with Y59, as well as a cation-Pi interaction with K65. This is in contrast to S9 of the CsEd toxin (Table S1). When analyzing the area of the structure where Y9 is located, it is observed that the temperature B factors are lower for these three residues S8, Y9, and T10 (Table S3). These data suggest that the contacts established by Y9 stabilize the corresponding area, which contributes to an increase in the affinity of scFv 10FG2 toward CsEM1a toxin.

In complexes made up of scFv LR-CsEd and scFv LR-CsEM1a, the average number of hydrogen bonding contacts throughout the dynamics was 13.1 for the CsEd toxin, while for the CsEM1a toxin, it was 8.79. These results indicated that scFv LR establishes a better interaction with CsEd. The MD (see Table S2) shows several hydrogen bonding contacts of the N31 of the scFv LR with the residues E15, K13, and N10 (Figure 3d), which do not occur with the CsEM1a toxin (Figure 3e). A hydrogen bond with CsEd toxin via E15 with A33 of LR can also be observed. Another residue of the CsEd toxin that interacts with the scFv LR is S54, while for CsEM1a, it is R27. These differences between the two toxins influence the variability in the affinities of LR for them, where the affinity for CsEd is higher. It is important to note that these CsEd toxin residues, with the exception of N10, are found in both CsEM1a and Cn2 toxin. The affinity of LR is significantly higher for the Cn2 toxin (KD = 1.12 × 10^−10^ M, and T_R_ = 333 min) [12]. This toxin, like CsEM1a, does not show contacts with N31 of the scFv LR; however, Cn2 establishes salt-bridge-type contacts through its D7 with the R53 of the scFv (see Table S2). These contacts are not seen with CsEd or CsEM1a toxins. Salt bridges have a higher energy than hydrogen bonds in general, which could explain the difference in affinity for the Cn2 toxin compared to the other two toxins studied. The observation that the same residues present in the three toxins do not make the same contacts with the scFv LR or 10FG2 suggests that there are additional factors that contribute to the observed differences in affinities and in the MD themselves. A possible explanation can be associated with the differences between toxins at the sequence level (91% identity between CsEd and CsEM1a toxins and 88% between Cn2 and CsEd), which would determine different molecular dynamics of adaptation between these toxins and the scFvs.

## 4. Conclusions

scFvs LR and 10FG2, whose characteristics have been previously published, are strong candidates to form part of an alternative broad-spectrum antivenom against scorpion sting in Mexico. In this work, we report how scFv 10FG2 neutralizes the effect of the venom of *C. sculpturatus*, and although scFv LR does not completely neutralize it, the combination of both showed a complementary effect since the efficiency with which the venom is neutralized was improved. The study of molecular interactions between scFvs and toxins revealed that many of the relevant contacts at the binding interfaces are maintained, so it was not necessary to perform affinity optimization of scFvs in order to neutralize *C. sculpturatus* main toxins. These observations allow to propose that in some cases, the mixture of these antibodies will make it possible to neutralize toxins and/or venoms from other Mexican scorpions and from neighboring countries where there are other species of the genus *Centruroides* bearing similar toxins.

## 5. Materials and Methods

### 5.1. Venom and Toxins

Venom of *C. sculpturatus* was acquired from the Spider Pharm and venom company from Santa Rita Foothills (SR). The lyophilized venom was diluted in tetra-distilled water and centrifuged at 14,000 rpm for 15 min at 4 °C. The insoluble material was discarded, whereas the toxin-containing supernatant was recovered and spectrophotometrically quantified (λ = 280 nm). A total of 40 mg of venom was fractionated and the toxins isolated following the procedure described in [5] to obtain the CsEM1a and CsEd toxins

### 5.2. Expression of scFvs 10FG2 and LR

Protein expression and purification of each sequence was carried out using the pSyn1 plasmid and in *E. coli* TG1, as described previously [10]. The scFvs were always kept in 1x PBS buffer (137 mM NaCl, 2.7 mM KCl, 8 mM Na_2_HPO_4_, 1.5 mM KH_2_PO_4_, pH 7.4). The protein concentration was determined spectro-photometrically at λ = 280 nm.

### 5.3. Surface Plasmon Resonance Recognition and Affinity Determinations

For these assays, we used chips CM5, the Amine Coupling Kit (Biacore), and a Biacore biosensor system (Biacore X, Uppsala, Sweden). For each toxin, 250 ng was dissolved in 100 μL of 10 mM 2-(N-morpholino) ethanesulfonic acid (pH 6). A total of 10 μL of toxin solution was bound to cell 2 of the CM5 sensor chip previously activated at a flow rate of 5 μL min^−1^. Approximately 100 resonance units (RU) were coupled. After coupling, during the assays, the cell 1 (nothing bound) was used as a control. The protein solutions of scFvs were serially diluted in HBS-EP buffer (Biacore); 100 μL of samples of scFvs were injected over each chip (CsEM1a or CsEd coupled) at a flow rate 50 μL min^−1^. Biosensor measurements were performed at 25 °C. The scFv protein concentrations ranging from 0.5 nM to 180 nM were assayed. The delay phase lasted 1000 s. The chip surfaces were regenerated with 10 mM Glycine-HCl pH = 2. The kinetic constants were determined using the corresponding sensorgrams, which were corrected by subtracting the values from both the reference flow cell and the blank buffer injection. The Langmuir (1:1) model from BIA-evaluation software version 3.1 was used for kinetic constants determination.

### 5.4. Competition Assays by Means of Surface Plasmon Resonance

SPR binding assays to confirm that scFvs bind to different epitopes on toxins were performed. The sensor chip was prepared as described above. Three saturating amounts (30 µL of 0.5 mM) of scFv 10FG2 were consecutively injected onto a CsEM1a-coated chip at a rate of 20 µL min^−1^ in HBS-EP buffer, up to saturation of the available sites. Afterwards, 30 µL of the scFv LR at a 0.5 mM concentration were injected and the sensorgram analyzed. As controls, a sample of scFv LR recognizing CsEM1a were injected and compared with the competition.

### 5.5. Venom Neutralization Tests

#### 5.5.1. Mixed Test

To evaluate the neutralization activity against whole venom, groups of 6 CD1 female mice were used in most cases (except in 2 cases, 10 animals were used) by intraperitoneal injection, following the protocols approved by the Bioethics Committee of Instituto de Biotecnología of UNAM (Project number 413, Generation of a recombinant anti-venom against venomous scorpion stings). In a preliminary trial 1, an amount of venom equivalent to 1 LD_50_ or 2 LD_50_ (23 µg or 46 µg of venom) was mixed just with the scFv 10FG2 or scFv LR and the mix of scFvs 10FG2 plus LR with their respective controls (one LD_50_ or 2 LD_50_ of whole venom in 1X PBS buffer). Subsequently, the mixing tests were performed of scFv(s) and venom to several toxin:scFv molar ratios. These ratios were calculated relative to the main toxin in the venom. The LD_50_ of the venom of *C. sculpturatus* is ~23 µg/20 g of mouse [4], where toxins represent 9.6~10% of the total toxins. The amount corresponding to 2, 3, 4, or 5 LD_50_ of each venom was mixed with a fixed amount of scFv 10FG2 or 10FG2 plus LR. The mixture of venom and scFv(s) were pre-incubated at room temperature (~25 °C) for 30 min prior to their injection into the mice.

#### 5.5.2. Rescue Test

This experiment was performed for evaluate of the ability of scFv 10FG2 in combination with the scFv LR to rescue mice that were previously envenomed with 3 LD_50_ (69 µg of venom). A time span of 5–10 min was allowed to elapse before the mice were injected with both scFvs representing 1:5 toxin:scFv molar ratios of each one. The relative molar ratios were established assuming that 10% of venom corresponds to toxic components.

### 5.6. Modelling and Structural Analyses of scFv 10FG2-CsEM1a, scFv 10FG2-CsEd, scFv LR-CsEM1a, and scFv LR-CsEd Complexes

With the aim of exploring the structural basis of the neutralization of CsEM1a and CsEd toxins by scFv 10FG2 or scFv LR, models of this scFvs complexed with these two toxins were prepared based on the scFv RU1-Cn2-LR ternary complex structure model [17]. Additionally, the model of scFv 10FG2-Cn2 complex previously assembled in [11] was also used. Using the Maestro Program [23], 10FG2-Cn2 complex was modified by replacing the amino acids required to transform Cn2 toxin into CsEM1a or CsEd toxins according to the amino acid sequences shown in Figure 1. The three models (10FG2 or LR complexed with Cn2, CsEM1a, or CsEd toxins), were adjusted with the Protein Preparation Wizard module provided with the Maestro Program and a 15 15 Å buffered box of water with 0.15 M of NaCl was added by means of the System Builder module and adjusted to minimize the volume. The models were subjected to energy minimization procedures until 0.1 Kcal/mol/A was reached and then adjusted to a minimum of 2000 iterations, 3 LBFGS vectors, and a minimum of 20 SD (steepest descend) steps. The scFv-toxin complexes were submitted to MD simulation procedures, using the Desmond Program [24]. By using the Viparr utility provided with the Desmond program, the Charmm22star force field and the space water model force field were settled to all of the three systems. Then, they were submitted to MD on to the Desmond program [24] with the following settings: a MD simulation time of 100 ns; trajectory recording intervals of 10 ps (picoseconds) and five ps for energy recordings; NPT ensemble class was settled at a temperature of 300 K. The Langevin thermostat and barostat methods were used to control temperature and pressure, with 100 ps of relaxation time for both methods. We used an integration time step of two ps and Coulombic radius cut off of 9 Å (default value). A sample of twenty structural frames from each model complex was extracted at even intervals from the trajectories generated by MD simulations for the analysis of the interactions at the interphase between the scFvs 10FG2 or LR and the different toxins. From these samples, one of every 5000 frames was taken and submitted to the PIC (Protein Interactions Calculator) software using default values [25] and to PISA software [26] for analyses of the interface between the scFvs and each of the toxins evaluated.

## Figures and Tables

**Table 1:** Kinetic constants of the interaction of scFvs 10FG2 and LR with *C. sculpturatus* toxins.

scFv	Toxin	*k*on (M^−1^s^−1^) × 10^5^	*k*off (s^−1^) × 10^−4^	K_D_ (M)	T_R_ (min)
LR	CsEd	3.85	2.35	6.1 × 10^−10^	70.9
	CsEM1a	6.28	7.9	1.29 × 10^−9^	21
10FG2	CsEd	1.85	3.4	1.84 × 10^−9^	49
	CsEM1a	2.37	2.61	1.1 × 10^−9^	63

Molecular interactions were performed at 25 °C with a flow rate of 50 μL min^−1^. The affinity constants were calculated using Langmuir (1:1) models created by means of BIAevaluation 3.1 software. T_R:_ stands for time of residence.

**Table 2:** Preliminary assays of *C. sculpturatus* venom neutralization.

scFv(s)	LD_50_ of Venom	Molar Ratio	Survivors/Total
LR	1	1:10	9/10 **
LR	2	1:10	3/6 ***
10FG2	2	1:10	6/6 *
LR + 10FG2	2	1:5 of each scFv	6/6
Control	1	-	5/10 ***
Control	2	-	0/6 ***

The signs related to the effects of the toxic components of the venom are indicated and stand for: *, minimal (bristly hair and itching); **, middle (involuntary tail movement, abdominal contraction); ***, strong (salivation, shortness of breath, paralysis of the legs, death). The molar ratio of toxins and antibodies is established considering that the toxins correspond to ~10% of the venom. Controls of envenoming correspond to 1 LD_50_ and 2 LD_50_ of venom (23 µg and 46 µg/20 g of mouse). For the neutralization of 1 LD_50_ and 2 LD_50_, 87.4 µg and 174.8 µg of the corresponding scFv were used, respectively. In the case of the mix of LR and 10FG2 to neutralize 2 LD_50_ of venom, the amount of each of them was 87.4 µg/20 g of mouse.

**Table 3:** Formal evaluation of the neutralization of the venom of *C. sculpturatus*.

	Survivors/Total			
Mix assays scFv(s)	LD/Molar ratios (toxin:scFv)			
	2 LD_50_ 1:5	3 LD_50_ 1:3.3	4 LD_50_ 1:2.5	5 LD_50_ 1:2
10FG2	6/6	6/6	6/6 *	6/6 *
Comb. LR+10FG2	-	-	6/6	6/6
Control 2 LD_50_	0/6			
Comb. LR+10FG2	6/6			
Control	0/6			

Comparison of neutralization capacity between scFv 10FG2 alone and combined with scFv LR in the venom mix assay. Molar ratios (toxins: scFv 10FG2) = 1:5, 1:3.3, 1:2.5, and 1:2. Molar ratios (toxins: scFv LR: scFv 10FG2) = 1:2.5:2.5 and 1:2:2. Controls of envenoming correspond to the administration of 2 LD_50_ of venom. *, minimal signs of envenoming (bristly hair and itching).

**Table 4:** Rescue test from 3 LD_50_ of the venom of *C. sculpturatus*.

	Survivors/Total
Comb. LR+10FG2	6/6
Control	0/6

Rescue of envenomed mice with 3 LD_50_ of venom by a mix of both scFvs using a toxin: scFv molar ratio of 1:5:5 (toxin: scFv 10FG2: scFv LR). Controls of envenoming correspond to the administration of 3LD_50_ of venom.

## Data Availability

The data presented in this study are available in this article and in the Supplementary Materials.

## Supplementary Materials

The following are available online at https://www.mdpi.com/article/10.3390/toxins13100708/s1. Figure S1: Competition analysis by SPR of scFvs 10FG2 and LR for interaction with toxin CsEd, Table S1: Comparison of interactions between scFv 10FG2 and the indicated toxins, Table S2: Comparison of interactions between scFv LR and the indicated toxins, Table S3: Temperature B factors of the first 20 residues of toxins CsEM1a and CsEd and their difference during interaction with scFv 10FG2.

## References

[1] Gonzalez-Santillan E., Possani L.D. (2018). North American scorpion species of public health importance with a reappraisal of historical epidemiology. Acta Trop..

[2] Kang A.M., Brooks D.E. (2017). Geographic Distribution of Scorpion Exposures in the United States, 2010–2015. Am. J. Public Health.

[3] Gummin D.D., Mowry J.B., Beuhler M.C., Spyker D.A., Brooks D.E., Dibert K.W., Rivers L.J., Pham N.P.T., Ryan M.L. (2020). 2019 Annual Report of the American Association of Poison Control Centers’ National Poison Data System (NPDS): 37th Annual Report. Clin. Toxicol..

[4] Riano-Umbarila L., Rodriguez-Rodriguez E.R., Santibanez-Lopez C.E., Guereca L., Uribe-Romero S.J., Gomez-Ramirez I.V., Carcamo-Noriega E.N., Possani L.D., Becerril B. (2017). Updating knowledge on new medically important scorpion species in Mexico. Toxicon.

[5] Carcamo-Noriega E.N., Olamendi-Portugal T., Restano-Cassulini R., Rowe A., Uribe-Romero S.J., Becerril B., Possani L.D. (2018). Intraspecific variation of Centruroides sculpturatus scorpion venom from two regions of Arizona. Arch. Biochem. Biophys..

[6] Gomez-Ramirez I.V., Riano-Umbarila L., Olamendi-Portugal T., Restano-Cassulini R., Possani L.D., Becerril B. (2020). Biochemical, electrophysiological and immunological characterization of the venom from Centruroides baergi, a new scorpion species of medical importance in Mexico. Toxicon.

[7] Olamendi-Portugal T., Restano-Cassulini R., Riano-Umbarila L., Becerril B., Possani L.D. (2017). Functional and immuno-reactive characterization of a previously undescribed peptide from the venom of the scorpion Centruroides limpidus. Peptides.

[8] Schiavon E., Pedraza-Escalona M., Gurrola G.B., Olamendi-Portugal T., Corzo G., Wanke E., Possani L.D. (2012). Negative-shift activation, current reduction and resurgent currents induced by beta-toxins from Centruroides scorpions in sodium channels. Toxicon.

[9] Boyer L., Degan J., Ruha A.M., Mallie J., Mangin E., Alagon A. (2013). Safety of intravenous equine F(ab’)2: Insights following clinical trials involving 1534 recipients of scorpion antivenom. Toxicon.

[10] Riano-Umbarila L., Juarez-Gonzalez V.R., Olamendi-Portugal T., Ortiz-Leon M., Possani L.D., Becerril B. (2005). A strategy for the generation of specific human antibodies by directed evolution and phage display. An example of a single-chain antibody fragment that neutralizes a major component of scorpion venom. FEBS J..

[11] Riano-Umbarila L., Gomez-Ramirez I.V., Ledezma-Candanoza L.M., Olamendi-Portugal T., Rodriguez-Rodriguez E.R., Fernandez-Taboada G., Possani L.D., Becerril B. (2019). Generation of a Broadly Cross-Neutralizing Antibody Fragment against Several Mexican Scorpion Venoms. Toxins.

[12] Riano-Umbarila L., Contreras-Ferrat G., Olamendi-Portugal T., Morelos-Juarez C., Corzo G., Possani L.D., Becerril B. (2011). Exploiting cross-reactivity to neutralize two different scorpion venoms with one single chain antibody fragment. J. Biol. Chem..

[13] Lopez-Giraldo A.E., Olamendi-Portugal T., Riano-Umbarila L., Becerril B., Possani L.D., Delepierre M., Del Rio-Portilla F. (2020). The three-dimensional structure of the toxic peptide Cl13 from the scorpion Centruroides limpidus. Toxicon.

[14] Pintar A., Possani L.D., Delepierre M. (1999). Solution structure of toxin 2 from centruroides noxius Hoffmann, a beta-scorpion neurotoxin acting on sodium channels. J. Mol. Biol..

[15] Saucedo A.L., del Rio-Portilla F., Picco C., Estrada G., Prestipino G., Possani L.D., Delepierre M., Corzo G. (2012). Solution structure of native and recombinant expressed toxin CssII from the venom of the scorpion Centruroides suffusus suffusus, and their effects on Nav1.5 sodium channels. Biochim. Biophys. Acta.

[16] Riano-Umbarila L., Rojas-Trejo V.M., Romero-Moreno J.A., Costas M., Utrera-Espindola I., Olamendi-Portugal T., Possani L.D., Becerril B. (2020). Comparative assessment of the VH-VL and VL-VH orientations of single-chain variable fragments of scorpion toxin-neutralizing antibodies. Mol. Immunol..

[17] Riano-Umbarila L., Ledezma-Candanoza L.M., Serrano-Posada H., Fernandez-Taboada G., Olamendi-Portugal T., Rojas-Trejo S., Gomez-Ramirez I.V., Rudino-Pinera E., Possani L.D., Becerril B. (2016). Optimal Neutralization of Centruroides noxius Venom Is Understood through a Structural Complex between Two Antibody Fragments and the Cn2 Toxin. J. Biol. Chem..

[18] Rodriguez-Rodriguez E.R., Olamendi-Portugal T., Serrano-Posada H., Arredondo-Lopez J.N., Gomez-Ramirez I., Fernandez-Taboada G., Possani L.D., Anguiano-Vega G.A., Riano-Umbarila L., Becerril B. (2016). Broadening the neutralizing capacity of a family of antibody fragments against different toxins from Mexican scorpions. Toxicon.

[19] Pucca M.B., Cerni F.A., Janke R., Bermudez-Mendez E., Ledsgaard L., Barbosa J.E., Laustsen A.H. (2019). History of Envenoming Therapy and Current Perspectives. Front. Immunol..

[20] Knudsen C., Ledsgaard L., Dehli R.I., Ahmadi S., Sorensen C.V., Laustsen A.H. (2019). Engineering and design considerations for next-generation snakebite antivenoms. Toxicon.

[21] Knudsen C., Casewell N.R., Lomonte B., Gutierrez J.M., Vaiyapuri S., Laustsen A.H. (2020). Novel Snakebite Therapeutics Must Be Tested in Appropriate Rescue Models to Robustly Assess Their Preclinical Efficacy. Toxins.

[22] Laustsen A.H., Maria Gutierrez J., Knudsen C., Johansen K.H., Bermudez-Mendez E., Cerni F.A., Jurgensen J.A., Ledsgaard L., Martos-Esteban A., Ohlenschlaeger M. (2018). Pros and cons of different therapeutic antibody formats for recombinant antivenom development. Toxicon.

[23] (2021). Schrödinger Release 2021-1.

[24] Bowers K.J., Chow D.E., Xu H., Dror R.O., Eastwood M.P., Gregersen B.A., Klepeis J.L., Kolossvary I., Moraes M.A., Sacerdoti F.D. Scalable algorithms for molecular dynamics simulations on commodity clusters. Proceedings of the SC ‘06: Proceedings of the 2006 ACM/IEEE Conference on Supercomputing.

[25] Tina K.G., Bhadra R., Srinivasan N. (2007). PIC: Protein Interactions Calculator. Nucleic Acids Res..

[26] Krissinel E., Henrick K. (2007). Inference of macromolecular assemblies from crystalline state. J. Mol. Biol..

