# Supplementary material for: Full Neutralization of Centruroides sculpturatus Scorpion Venom by Combining Two Human Antibody Fragments
Source: Toxins (Basel). 2021 Oct 6;13(10):708. doi: 10.3390/toxins13100708 (PMC8540208; doi:10.3390/toxins13100708)
Supplement: Supplementary file 1 [file toxins-13-00708-s001.zip › toxins-1374994-supplementary.pdf]

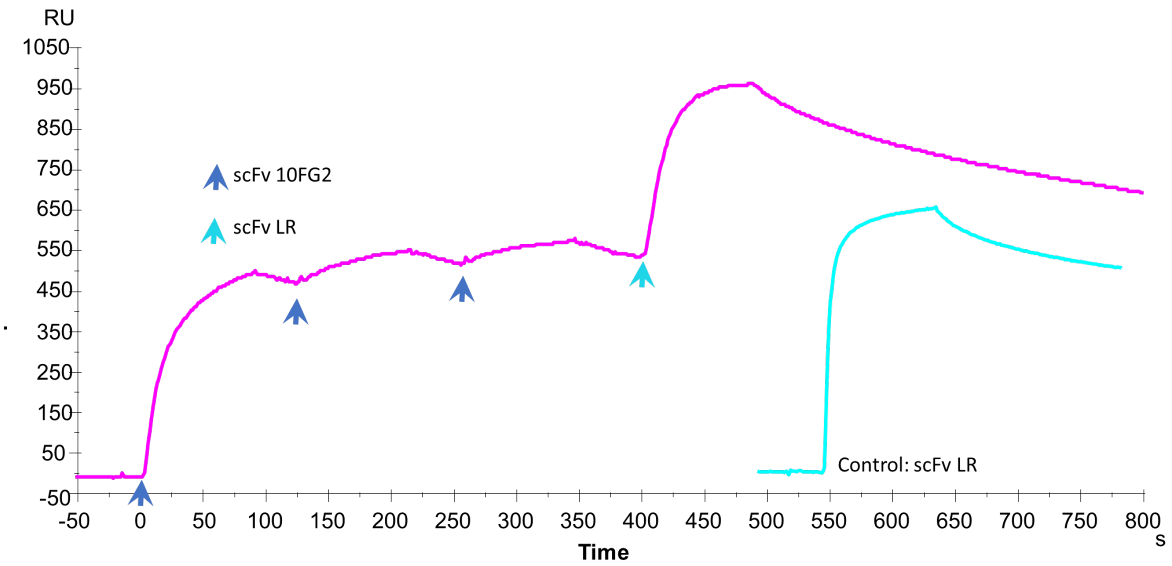

Figure S1. Competition analysis by SPR of scFvs 10FG2 and LR for interaction with toxin CsEd. Flow rate of 20 μL min^{-1} and a concentration of 500 nM of each scFv. RU: Resonance Units.

Table S1. Comparison of interactions between scFv 10FG2 and the indicated toxins

**10FG2 Residue [atom]**	**Cn2 Toxin Residue [atom] Å**[a]	**CsEd Toxin Residue [atom] Å**[a]	**CsEM1a Toxin Residue [atom] Å**[a]
**Hydrogen Bonds**			
**S31[O]**	K35[HZ] 1.95 (9)	K35[HZ2] 2.30 (1)	K35[HZ2] 2.30 (1)
S31[OG]	K35[HZ] 2.21 (5)		
Y53[HH]	**K30[O]** 1.76 (10)	**R30[O]** 1.73 (11)	**R30[O]** 1.87 (10)
G54[H]	Q31[OE1] 2.40 (2)	Q31[OE1] 231 (8)	Q31[OE1] 2.39 (3)
**G55[H]**		Q31[OE1] 2.19 (1)	
**G56[H]**	Q31[OE1] 2.33 (4)	Q31[OE1] 2.36 (6)	Q31[OE1] 2.32 (3)
Y59[OH]	Q32[HE22] 2.25 (4)	Q32[HE22] 2.16 (2)	Q32[HE22] 2.37 (1)
Y59[OH]	**K8[H]** 2.26 (3)	**K8[H]** 2.29 (4)	**S8[H]** 2.13 (3)
Y59[OH]		Y14[HH] 1.96 (1)	
Y59[HH]	Q32[OE1] 1.78 (6)	Q32[OE1] 1.80 (9)	Q32[OE1] 1.99 (6)
Y59[HH]	D7[OD2] 2.05 (2)		N7[OD1] 2.03 (1)
**Y60[O]**	K8[HZ] 1.91 (2)	K8[HZ] 2.24 (2)	
**Y60[O]**	K8[HZ3] 2.04 (1)		
**Y60[H]**			Y9[OH] 2.28 (6)
R101[HH]		Q54[OE1] 2.13 (2)	
D102[OD2]	**K35[H]** 1.90 (10)	**K35[H]** 2.05 (6)	**K35[H]** 2.03 (2)
D102[OD1]		**K35[H]** 2.10 (2)	**K35[H]** 2.11 (3)
D102[OD2]	K35[HZ] 1.77 (11)	K35[HZ] 1.70 (5)	K35[HZ] 1.70 (3)
D102[OD]	Y52[HH] 1.69 (9)	Y52[HH] 1.80 (11)	Y52[HH] 1.69 (9)

D102[OD1]		K8[HZ3] 1.79 (1)	
D102[OD1]		**K8[H]** 1.94 (1)	
C103[SG]		**Q32[O]** 3.90 (1)	**Q32[O]** 3.70 (3)
**L104[H]**	**Q32[O]** 1.93 (11)	**Q32[O]** 2.00 (11)	**Q32[O]** 2.07 (10)
**L105[O]**	**I56[H**] 2.04 (9)	**V56[H]** 2.16 (8)	**V56[H]** 2.13 (10)
**L105[H]**	**Q32[O]** 2.43 (3)		**Q32[O]** 2.24 (5)
S107[OG]	**Q54[O]** 3.36 (2)	**Q54[O]** 3.24 (5)	**Q54[O]** 3.56 (7)
S107[OG]	Q54[HE21] 2.04 (3)		
**S107[H]**	**Q54[O]** 2.11 (11)	**Q54[O]** 1.94 (11)	**Q54[O]** 2.10 (11)
D108[OD]	Q54[HE21] 2.17 (3)	Q54[HE21] 1.93 (2)	
T172[OG1]	Q54[HE22] 2.07 (4)		
**D233[O]**	N62[HD2] 2.00 (3)	N62[HD21] 2.31 (3)	N62[HD21] 2.07 (2)
S234[OG]	N62[HD22] 2.06 (1)		
T235[OG1]		**N62[O]** 3.85 (3)	
**T235[O]**	K8[HZ3] 2.20 (3)	K8[HZ] 2.29(6)	
**T235[O]**		K63[HZ3] 2.12 (2)	
**L236[O]**	K8[HZ] 2.04 (5)	K8[HZ1] 2.01 (6)	
Salt Bridges			
D62[OD2]	K8[NZ] 3.50 (4)		
D102[OD2]	K35[NZ] 2.73 (10)	K35[NZ] 2.73 (6)	K35[NZ] 2.67 (2)
**Hydrophobic Interactions within 5Ǻ**			
Y59			Y9
Y60			Y9
L105	Y33	Y33	Y33
L105	L5	L5	L5
L105	V6		
L105	I56	V56	V56
L105	V6	V6	V6
W231	I56		
L236	I56		
L236	L60	L60	L60
L236	P61		
**Aromatic-Aromatic Interactions within 4.5 and 7 Angstroms [Distance, Angle]**			
Y59			Y9[5.16, 103.02] (11)
**Aromatic-Sulfur Interactions within 5.3Ǻ [Distance, Angle]**			
C106	Y52[5.08, 70.31] (6)	Y52[4.57, 44.53] (8)	Y52[4.92, 55.77] (8)
C103	Y52[5.11, 69.20] (2)	Y52[4.87, 52.39] (9)	Y52[5.02, 59.82] (5)
C103	Y33[5.02, 98.77] (2)	Y33[5.22, 97.54] (1)	Y33[5.22, 97.66] (1)
**Cation-Pi Interactions within 6Ǻ [Distance, Angle]**			
R101	Y52[4.97, 22.28] (8)	Y52[5.23, 18.68] (7)	Y52[5.29, 17.95] (10)
Y53	K35[5.38, 164.59] (9)	K35[5.12, 163.28] (5)	K35[5.19, 152.31] (6)

Y32	K35[5.93, 37.01] (2)		
W47	K8[5.87, 86.41] (3)	K8[5.88, 76.83] (3)	
Y59	K8[5.35, 27.64] (4)	K8[5.50, 22.82] (3)	
K65			Y9[4.78, 23.00] (10)
W231	K8[5.77, 70.82] (1)	K8[5.77, 70.79] (1)	

[a] Average distance in different frames of the sample taken from MD. The number in parentheses indicates how many times the contact was observed in the sample taken from MD. Bold letters indicate interactions with the main chain of the indicated residue.

Table S2. Comparison of interactions between scFv LR and the indicated toxins

**LR Residue [atom]**	**Cn2 Toxin Residue [atom] Å[a]**	**CsEd Toxin Residue [atom] Å[a]**	**CsEM1a Toxin Residue [atom] Å[a]**
Hydrogen Bonds			
N31[O]		E15[H] 2.15 (15)	
N31[HD2X]		K13[O] 2.05 (17)	
N31[OD1]		N10[HD2X] 1.97 (21)	
N31[HD22]		N10[OD1] 2.17 (3)	
Y32[HH]	**S66[O]** 1.79 (4)	**S66[O]** 1.81 (1)	**N66[O]** 1.78 (1)
Y32[HH]	S66[OG] 1.77 (1)		
Y32[OH]	C65[SG] 3.41 (1)	C65[SG] 3.64 (3)	
Y32[HH]	C65[O] 2.06 (2)		
Y32[OH]		C12[SG] 3.46 (3)	
A33[H]		E15[OEX] 1.86 (19)	
H35[NE2]	E15[OE2] 3.66 (1)	E15[OE2] 3.66 (2)	E15[OE2] 3.68 (4)
R53[HH1X]	**D7[ODX]** 1.85 (24)	**N7[OD1]** 1.91 (4)	**N7[OD1]** 2.20 (2)
R53[HH11]	K13[O] 2.05 (15)		
R53[O]	Y24[HH] 1.65 (3)	Y24[HH] 1.88 (1)	Y24[HH] 1.64 (1)
R53[HH12]	C12[O] 2.13 (1)		
R53[HH22]		E28[OE2] 1.83 (1)	
R53[HH21]		Y14[OH] 1.98 (2)	
R53[HH12]			Y14[OH] 2.05 (1)
R53[O]		R27[HH22] 2.27 (3)	
S54[OG]	R27[HH22] 2.18 (6)		
S54[O]	R27[HH22] 1.95 (3)	R27[HH22] 2.02 (11)	
S55[O]	R27[HHX2] 1.99 (4)	R27[HHX2] 1.79 (5)	
G56[O]			R27[HH22] 2.33 (3)
D57[ODX]	L17[H] 1.84 (21)	L17[H] 1.83 (20)	L17[H] 1.89 (19)
D57[ODX]	K18[H] 2.03 (19)	K18[H] 2.10 (19)	K18[H] 1.99 (18)
D57[OD1]	K18[HZX] 1.71 (4)	K18[HZX] 1.71 (16)	K18[HZX] 1.71 (19)
D57[OD1]		K18[NZ] 2.67 (1)	

D57[ODX]	N22[HD22] 2.00 (19)	N22[HD22] 1.91 (15)	N22[HD22] 1.88 (14)
D57[O]			Y24[HH] 1.61 (1)
I58[O]	K18[HZX] 1.91 (2)	K18[HZX] 1.81 (14)	K18[HZX] 1.94, (12)
D59[OD2]	K18[HZ3] 1.69 (1)		
D59[OD2]	K18[HZ1] 1.60 (1)	K18[HZ1] 2.03 (1)	
R98[HH22]	S66[O] 2.41 (1)		
G100[H]	E15[OE2] 2.17 (1)	E15[OEX] 2.15 (8)	E15[OEX] 2.16, (8)
F101[H]	E15[OE1] 1.18 (12)	E15[OEX] 1.99 (9)	E15[OEX] 2.01 (14)
G102[H]	E15[OEX] 2.05 (14)	E15[OEX] 2.11 (10)	E15[OE2] 2.14 (13)
R163[HH22]	Y42[OH] 1.78 (1)		
R163[HH11]	Y42[OH1] 2.10 (1)		
Y165[HH]	Y4[OH] 2.32 (1)		Y4[OH] 2.09 (4)
Y165[OH]	Y42[HH] 2.07 (1)		
R229[HHX2]	E15[OEX] 1.88 (34)	E15[OEX] 1.96 (38)	E15[OEX] 1.87 (31)
R229[HH21]	C16[O] 2.28 (2)	C16[O] 2.33 (1)	C16[O] 2.50 (1)
Salt Bridges			
H35[NE2]	E15[OEX] 3.78 (4)	E15[OEX] 3.66 (2)	E15[OEX] 3.74 (9)
R53[]NH1]	D7[OD2] 3.28 (11)		
R53[]NH1]	D7[OD1] 3.87 (5)		
R53[NH2]	D7[OD2] 2.79 (13)		
R53[NH2]	D7[OD1] 3.18 (10)		
R53[]NH1]		E28[OE2] 2.81 (1)	
R53[]NH2]		E28[OE2] 2.81 (1)	
D57[ODX]	K18[NZ] 2.73 (7)	K18[NZ] 2.81 (18)	K18[NZ] 2.71 (19)
R98[NHX]	**S66[O]** 3.23 (1)	**S66[O]** 3.79 (1)	**N66[O]** 3.58 (2)
R229[NHX]	E15[OEX] 3.16 (39)	E15[OE2] 2.92 (39)	E15[OE2] 2.81 (37)
Hydrophobic Interactions within 5Å			
A33	L17 (9)	L17 (18)	L17 (10)
W47	L17 (4)	L17 (11)	L17 (12)
I51			L17 1
F101	A45 (2)	A45 (19)	A45 (13)
F101		F44 (8)	
F101			W58 (1)
Y165	Y4 (1)		Y4 (4)
Y165	Y42 (14)	Y42 (3)	Y42 (11)
Y165	A43 (19)	A43 (13)	A43 (19)
Y224	F44 (20)	F44 (20)	F44 (18)
Y224	A43 (4)	A43 (2)	A43 (3)
Y226	L19 (2)	L19 (1)	L19 (2)

Aromatic-Aromatic Interactions within 4.5 and 7 Angstroms [Distance, Angle]			
W47		F44 [6.80; 100.67] (1)	F44 [6.74; 126.53] (1)
F101		W58 [6.97; 109.02] (1)	W58 [6.05; 100.86] (1)
Y165	Y42[6.47; 86.29] (13)	Y42 [6.27; 87.96] (4)	Y42 [6.47; 84.29](13)
Y65			Y4[6.82; 80.36] (1)
Y224	F44[6.77; 105.36] (6)	F44[6.93 ; 110.57] (1)]	F44 [6.87; 119.96] (2)
Aromatic-Sulfur Interactions within 5.3Å [Distance, Angle]			
Y32	C12[5.01; 32.08] (3)	C12[5.16 ; 56.54] (1)	
Y32	C65[4.74; 48.75] (17)	C65[4.80 ; 50.80] (1)	C65 [4.55; 61.04] (1)
F101	C65[5.28; 72.12] (17)	C65[4.52 ; 90.00] (2)	C65 [4.62; 70.78] (7)
F101	C12[5.04; 56.73] (1)		C12 [4.92; 57.80] (2)
Cation-Pi Interactions within 6Å [Distance, Angle]			
R53	Y14[3.92; 17.28] (20)	Y14 [5.23; 126.92] (17)	Y14 [5.03; 135.42](9)
F101	K13[5.55; 53.05] (7)	K13 [5.08; 33.29] (18)	K13 [5.41; 46.96] (8)
Y165			K13[5.86; 90.18] (1)
R225		Y42 [5.51; 99,76] (3)	Y42 [5.15; 71.17](5)
R229	F44[4.58; 40.18] (19)	F44 [4.50; 28.30] (20)	F44 [4.34; 30.66](19)

[a] Average distance in different frames of the sample taken from MD.

The number in parentheses indicates how many times the contact was observed in the sample taken from MD.

Bold letters indicate interactions with the main chain of indicated residue.

Table S3. Temperature B factors of the first 20 residues of toxins CsEM1a and CsEd and their difference during interaction with scFv 10FG2

		**CsEM1a**		**CsEd**	**Difference**
Residue		B factor		B factor	CsEM1a-CsEd
1	K	167.6296969387	K	35.302643674	132.3270532647
2	E	18.054839999	E	19.262340288	-1.207500289
3	G	5.0506361493	G	4.4643463912	0.5862897581
4	Y	5.9322012627	Y	5.2696473751	0.6625538876
HH 5	L	19.9735798205	L	16.7065914745	3.2669883459
HH 6	V	14.8477939047	V	15.6693531253	-0.8215592207
C 7	N	36.0077170784	N	39.8229365656	-3.8152194872
CC 8	S	32.8495186243	K	40.3840991739	-7.5345805496
C 9	Y	25.3281339078	S	49.2956494218	-23.9675155141
10	T	19.321280032	N	34.4138114961	-15.0925314641
11	G	8.2076367038	G	10.9841040141	-2.7764673103
12	C	7.7108895193	C	10.4611618013	-2.750272282
13	K	35.2774719618	K	38.9140703605	-3.6365983987
C 14	Y	57.3110461853	Y	49.485018431	7.8260277543
15	E	38.5993317058	E	35.0650328579	3.5342988479
16	C	5.9607549183	C	5.1599951324	0.8007597859
17	L	50.2558412015	L	53.5181320732	-3.2622908717
18	K	73.2126242526	K	80.8775249039	-7.6649006513
19	L	25.1397119297	L	20.7111697703	4.4285421594
20	G	8.0152927149	G	6.8558417737	1.1594509412
